# WaveletUMamba: a frequency-enhanced state space model for extraction of multi-stage celery planting areas from UAV imagery

**DOI:** 10.3389/fpls.2026.1897082

**Published:** 2026-07-28

**Authors:** Rui Li, An Huang, Xue Ding, Shuangyun Peng, Yanjv Yu, Yezhou Xiang

**Affiliations:** 1 School of Information Science and Technology, Yunnan Normal University, Kunming, China; 2 Faculty of Geography, Yunnan Normal University, Kunming, China; 3 Key Laboratory of Resources and Environmental Remote Sensing for Universities in Yunnan, Kunming, China; 4 Center for Geospatial Information Engineering and Technology of Yunnan Province, Kunming, China; 5 Engineering Research Center of Computer Vision and Intelligent Control Technology, Yunnan Provincial Department of Education, Kunming, China

**Keywords:** celery, Mamba, multi-growth stages, planting area extraction, UAV remote sensing, wavelet transform

## Abstract

In the task of extracting celery cultivation areas across different growth stages from high-resolution UAV remote sensing imagery, the original Mamba model exhibits a bias toward low-frequency information during state-space modeling and responds relatively poorly to high-frequency details such as boundaries and textures, thereby affecting its ability to characterize fine-grained spatial structures in complex scenes. To address this issue, this paper proposes a WaveletUMamba network that integrates frequency-domain enhancement with state-space modeling. First, a Low-Frequency Masking High-Frequency Enhancer (LFM-HFE) was designed to enhance high-frequency information—such as edges and textures—based on two-dimensional Haar wavelet decomposition, thereby compensating for the state-space model’s shortcomings in modeling local details. Second, a Dual-Gated Frequency-Spatial State-Space Module (DG-SSM) was constructed to achieve the synergistic fusion of high-frequency detail features with the global state-space modeling process, thereby enhancing the model’s ability to represent complex spatial structures. Finally, to mitigate category confusion caused by similar textural and spectral features across different growth stages, a Context-Guided Feature Supervision module (CGFS) was designed to enhance the discriminative power and consistency of intermediate layer features through global semantic constraints. Experimental results show that this method achieves 88.70% and 93.90% for the mIoU and mF1 metrics, respectively—representing improvements of 1.07% and 0.30% over the second-best model, UNetMamba—while striking an optimal balance between extraction accuracy and computational efficiency. Ablation experiments and visualization results further validate the effectiveness of each module. The WaveletUMamba framework proposed in this paper offers a new methodological approach for the precise extraction of crop planting areas across multiple growth stages in complex open-field agricultural environments, while also providing a technical solution for the practical application of UAV remote sensing technology in extracting crop planting areas.

## 1 Introduction

Celery (Sowbhagya, 2014) is an important vegetable with both medicinal and culinary uses, and it is widely cultivated and consumed around the world. China is a major producer and consumer of celery globally. As a high-value-added vegetable, it is widely cultivated in major highland vegetable-producing areas such as Tonghai County, Yuxi City, Yunnan Province. As the scale of celery cultivation continues to expand, traditional methods of monitoring plant growth—which rely on manual field sampling and fixed-point observations—suffer from drawbacks such as high labor costs, limited sampling scope, poor timeliness, and a high degree of subjectivity. These methods struggle to meet the demands for large-scale, high-throughput, and dynamic growth monitoring in open fields.

Low-altitude UAV remote sensing technology (Osco et al., 2021), with its advantages of high spatial resolution, operational flexibility, short data acquisition cycles, low monitoring costs, and non-destructive detection, has become an important technical tool for extracting phenotypic information from agricultural crops (Li et al., 2026; Zheng et al., 2026) and monitoring growth trends across multiple time phases (Omia et al., 2023), providing an efficient method for acquiring data on celery cultivation areas at different growth stages. However, in the complex environment of open-field farmland, achieving high-precision identification of celery cultivation areas throughout the entire growth cycle still poses numerous challenges. First, remote sensing imagery is easily interfered with by background factors such as bare soil, weeds, shadows, and other similar crops, which reduces the distinction between target and background features. Second, celery takes a long time to grow, and plants at different stages of growth have very similar spectral, textural, and morphological traits. During the seedling stage, canopy cover is low and the proportion of bare soil is high, making the target susceptible to interference from complex backgrounds. As plants enter the early leaf development stage and rapid leaf development stage, the canopy gradually closes, further reducing spectral and morphological differences between adjacent growth stages, which can easily lead to feature overlap and class confusion. Furthermore, open-field farmland contains both large-scale, contiguous planting areas and a large number of scattered, small-scale plots. This significant spatial heterogeneity and the complexity of plot boundaries further exacerbate the difficulty of extraction and place higher demands on the model. These factors have led to widespread issues such as blurred boundaries, category confusion, and the failure to detect small-scale targets during the extraction of celery cultivation areas at different growth stages. These challenges make it difficult to achieve stable, high-precision extraction of celery cultivation information throughout the entire growth cycle, thereby limiting the application of UAV remote sensing technology in celery growth monitoring and precision agriculture.

Currently, extensive research has been conducted on crop information extraction using remote sensing. Early methods primarily relied on manually extracting low-level visual features such as the Gray-level Co-occurrence Matrix (Gadkari, 2004) and the Histogram of Oriented Gradients (Wang et al., 2019), combined with traditional machine learning methods such as support vector machines and random forests to perform pixel-by-pixel classification. Such methods are limited by their feature representation capabilities. They struggle to effectively address changes in illumination and spectral interference from non-crop objects in complex agricultural scenes, and they lack the ability to characterize subtle feature differences across different growth stages of celery. Consequently, they fail to meet the demand for precise feature extraction in complex agricultural scenarios involving crop cultivation areas spanning multiple growth stages.

With the rapid development of deep learning technology, neural network-based semantic segmentation models (Luo et al., 2024) have made significant progress in remote sensing-based crop information extraction. CNNs (Alzubaidi et al., 2021) extract features through layer-by-layer convolution and use pooling operations to progressively reduce the size of feature maps and expand the receptive fields of neurons. They excel at local feature extraction and are widely used in crop cultivation area extraction tasks. Typical models include U-Net (Ronneberger et al., 2015) and the DeepLab (Chen et al., 2018a) series. These methods significantly improve the accuracy and efficiency of crop area extraction through multiscale feature learning. For example, Zhang et al. (2023) improved the extraction accuracy of winter wheat cultivation areas while reducing the number of model parameters by introducing a lightweight MobileNetV2 backbone network and the CBAM attention mechanism into the DeepLabv3+ framework. However, the local connectivity mechanism of convolutional operations imposes inherent limitations on their receptive fields, making it difficult for models to establish long-range spatial contextual dependencies. When processing high-resolution UAV imagery of complex agricultural landscapes, CNN models are prone to semantic discontinuities, leading to localized fragmentation and incorrect boundary segmentation in the extraction results.

To address the limitations of CNNs in global modeling, the Transformer (Khan et al., 2022) architecture has gradually been introduced into remote sensing tasks for crop cultivation information extraction. By calculating the similarity of all spatial locations in a feature sequence, it can directly capture global contextual information, thereby enhancing target recognition capabilities in complex scenes. For example, Wang et al (Wang et al., 2024). improved the SegFormer model by introducing a multi-scale feature fusion network and a coordinate attention mechanism, significantly enhancing the accuracy of winter wheat cultivation area extraction under complex terrain conditions. Yan et al. (2023) proposed the ETUnet network, which combines dilated convolutions with the Transformer’s self-attention mechanism to achieve precise identification of rice paddies at different growth stages in UAV imagery. However, the computational complexity of the standard Transformer’s self-attention mechanism is O(N²). As the number of tokens in high-resolution remote sensing images rapidly increases, the computational load and GPU memory overhead grow quadratically. In the context of UAV remote sensing imagery, this high complexity not only increases the costs of model training and inference but also limits deployment efficiency on resource-constrained platforms.

In recent years, the Mamba architecture (Gu and Dao, 2024), based on state-space models, has gradually garnered attention in computer vision tasks. The Mamba model is capable of modeling global features and efficiently capturing temporal information with lower computational complexity, demonstrating strong application potential in UAV remote sensing tasks. Currently, researchers have applied the Mamba architecture to crop cultivation information extraction tasks. For example, Luo et al. (2025) proposed MASNet, a parallel dual-encoding network that fuses CNNs, Transformers, and Mamba and, by incorporating a multi-task learning mechanism, improved the robustness of complex farmland parcel extraction. Ge et al. (2025) proposed the lightweight XM-UNet model, which combines Mamba’s selective scanning mechanism with CNNs to achieve an overall accuracy of 94.26% in the rice distribution recognition task. Although CNNs and Transformers excel in local feature extraction and global modeling, respectively, the former is limited by receptive field size, while the latter faces issues of high computational complexity. The Mamba architecture (Bansal et al., 2024), through its selective scanning mechanism, effectively balances global perception and computational efficiency. This provides a new research approach to address the problems of blurred boundaries and confusion between growth stages in the fine-grained extraction of celery cultivation information across multiple growth stages.

However, Mamba’s linear scanning process inherently favors the propagation of low-frequency information (Liu et al., 2024; Chen et al., 2025). In the image domain, global context (such as the overall contours of objects) is primarily concentrated in low-frequency components, while high-frequency components (such as edges, textures, and noise) mostly represent local details. When performing global feature aggregation, Mamba smooths and averages local features, which results in a significant enhancement of the low-frequency components in its output feature maps, while high-frequency details are suppressed. Kim (Kim et al., 2025) and colleagues confirmed through spectral analysis that Mamba exhibits typical low-pass filtering characteristics in feature processing. This inherent structural bias triggers a noticeable “oversmoothing” phenomenon, causing high-frequency information to continuously attenuate and local features to become homogenized, thereby weakening the model’s ability to capture fine spatial details. When processing high-resolution UAV imagery, this low-pass filtering characteristic tends to smooth out local high-frequency structures such as texture details and boundary information, leading to blurred boundaries and loss of detail in fine-grained segmentation tasks. In the task of fine-grained extraction of celery cultivation information in complex open-field environments, high-frequency edge and texture features are precisely what is crucial for resolving issues such as classification confusion and boundary blurring.

The wavelet transform (Mallat, 1989; Li et al., 2020) can decompose features into different frequency subbands, thereby separating high-frequency edge and texture information from the mixed representation. This ability to decouple features across multiple scales in the frequency domain compensates for Mamba’s limitations in capturing high-frequency information. For example, Chen et al. (2026) utilized the discrete wavelet transform to decouple the frequency-domain components of an image, establishing a closed-loop interactive mechanism in which low-frequency components guide high-frequency generation, while high-frequency feedback corrects low-frequency structures. This effectively suppressed false textures and structural distortions while enhancing the restoration of fine-grained details. The collaborative modeling of Mamba and the wavelet transform preserves Mamba’s efficient global modeling capabilities while accurately restoring and enhancing high-frequency detail information, providing a new research approach to address the issues of blurred boundaries and confusion during the growing season in fine-grained extraction tasks for celery cultivation areas.

To address the aforementioned technical bottlenecks, this paper focuses on large-scale field cultivation of celery and, based on low-altitude UAV remote sensing imagery, proposes WaveletUMamba—a model for extracting detailed information throughout the entire growth cycle of celery that integrates frequency-domain enhancement with state-space modeling. While retaining Mamba’s efficient global modeling capabilities, this method effectively enhances the representation of local high-frequency details and improves classification accuracy across different growth stages by introducing frequency-domain enhancement and context-based semantic guidance. The main innovations and contributions of this study are as follows:

1. Proposal of the High-Frequency Feature Enhancer (LFM-HFE). To mitigate the tendency of the Mamba architecture to prioritize low-frequency information in state space modeling, LFM-HFE performs a frequency-domain decomposition of features using the Haar discrete wavelet transform. It focuses on enhancing high-frequency components through reconstruction and refinement, which helps improve the representation of boundary, texture, and fine-grained structural details.
2. We constructed a frequency-domain-enhanced state-space module (DG-SSM). To jointly model local fine-grained information and global contextual information, this module runs the LFM-HFE branch and the Mamba branch in parallel via a dual-track approach. It employs a spatial–channel dual attention mechanism as a gate to achieve adaptive noise filtering and feature calibration, significantly enhancing the ability to characterize regions at the boundaries between adjacent growth stages and small-scale objects.
3. Design of the Context-Guided Feature Supervision Module (CGFS). By introducing global semantic priors to provide guided supervision for deep features, this module supplies optimization signals with greater discriminative consistency during training, effectively alleviating the semantic confusion caused by the high spectral and textural similarity among celery classes across different growth stages.
4. We constructed a high-resolution UAV-based celery dataset spanning multiple growth stages and systematically validated the effectiveness of WaveletUMamba based on this dataset. Experimental results demonstrate that this method effectively improves the accuracy of cultivation area extraction while maintaining linear computational complexity, providing a viable technical approach for fine-grained crop monitoring in complex agricultural scenarios.

## 2 Related work

With the rapid development of high-resolution remote sensing technology, research on deep learning-based crop cultivation area extraction has made significant progress. Based on differences in network architecture and feature modeling mechanisms, existing remote sensing semantic segmentation methods can be broadly classified into three categories: methods based on convolutional neural networks (Alzubaidi et al., 2021), methods based on the Transformer architecture (Khan et al., 2022), and emerging methods based on state-space models (Gu and Dao, 2024). This paper conducts a qualitative comparative analysis of these three categories of methods with a focus on crop cultivation area extraction tasks. A review and summary of the relevant literature are presented in **Table 1**.

**Table 1:** Qualitative comparative analysis of methods for extracting crop-growing areas.

Method	Model/approach	Research data	Research task	Key contribution
CNN-based methods	Zhang et al. (2023) proposed an improved lightweight DeepLabv3+-based model.	Multispectral satellite imagery (Sentinel-2, GF-6) and RGB imagery.	Winter wheat planting area extraction.	Reduced model parameters while improving recognition accuracy.
	Jiang et al. (2025) constructed CNN-LSTM and Bi-LSTM models incorporating NDVI temporal features	Sentinel-2 time-series data.	Multi-crop planting area extraction.	Achieved an extraction accuracy of 96.48%.
	Kuang et al. (2023) proposed a hybrid method combining stacked autoencoder-based dimensionality reduction with CNN classification.	Multi-source satellite data.	Crop area prediction.	Effectively mitigated performance degradation in high-dimensional multi-source data, achieving 98.57% overall accuracy.
	Du et al. (2019) applied DeepLabv3+ within the TensorFlow framework for automatic mapping using high-resolution RGB imagery.	High-resolution RGB remote sensing imagery.	Smallholder planting area extraction.	Demonstrated the superiority of semantic segmentation in delineating small, irregular, and fragmented plots.
	Zhao et al. (2022) enhanced the U-Net by embedding CBAM attention modules in both encoder and decoder stages.	GF-6 and Sentinel-2 imagery.	Winter wheat planting area extraction.	Significantly improved the accuracy of winter wheat planting area extraction.
	Huang et al. (2019) proposed an improved SegNet semantic segmentation network.	Sentinel-2 imagery.	Classification of Crop-Growing Areas.	It reduces computational costs while maintaining a slight improvement in classification accuracy.
Transformer-based methods	Wang et al (Wang et al., 2024). improved SegFormer by introducing a multi-scale feature fusion network and a coordinate attention mechanism.	Sentinel-2 imagery.	Extraction from Winter Wheat Growing Areas.	Improved the accuracy of identifying winter wheat-growing areas in complex terrain.
	Yan et al. (2023) proposed the ETUnet network by combining hole convolution with the CBAM attention module.	UAV imagery.	Rice Field Identification.	Achieves the accurate identification of rice paddies at different growth stages.
	Niu et al. (2022) proposed the HSI-TransUNet model.	UAV hyperspectral imagery.	Classification of Crop-Growing Areas.	Achieved an overall accuracy of 86.05%.
	Li et al. (2025) proposed a lightweight model, ARG-TR, based on the SegFormer architecture.	UAV multispectral dataset.	Rice anomaly detection.	Improved model accuracy while maintaining real-time inference efficiency.
	Gibril et al. (2023) evaluated multiple Vision Transformers (ViTs).	Multi-source high-spatial-resolution imagery.	Large-scale date palm recognition and extraction.	Achieved high accuracy with fewer parameters.
	Du et al. (2025) constructed a cascaded CNN–Transformer hybrid framework.	Multi-source remote sensing data.	Winter wheat yield estimation.	Significantly improved yield estimation accuracy.
	Han et al. (2025) proposed a dual-branch ConvFlow Transformer model.	UAV tobacco dataset.	Crop classification.	Effectively alleviated inter-class similarity issues.
Mamba -based methods	Luo et al. (2025) proposed MASNet, a parallel dual-encoder network integrating CNN, Transformer, and Mamba.	Solafune farmland dataset and Jilin-1 dataset.	Complex farmland extraction.	Improved robustness and geometric accuracy, achieving a peak mIoU of 72.24%.
	Ge et al. (2025) proposed a lightweight XM-UNet integrating Mamba selective scan (SS2D) with CNN.	Sentinel-1 SAR data (USA, Kenya, Vietnam).	Rice distribution mapping.	Achieved high overall accuracy with extremely low parameter count and improved interpretability.
	Fan et al. (2025) proposed a bidirectional spatiotemporal Mamba-UNet (BTSM-UNet).	Sentinel-2 time-series data (North China Plain).	Winter wheat classification.	Achieved the highest F1 score of 0.952 with improved efficiency and stability.

As shown in **Table 1**, current research on crop cultivation area extraction using deep learning has made significant progress.

In terms of research methods, studies on crop cultivation area extraction are primarily based on CNNs and Transformers. CNNs can effectively extract local spatial features through hierarchical feature learning and multiscale information fusion and have been widely applied in the extraction of crop cultivation areas from agricultural remote sensing imagery. However, due to their limited receptive fields, they struggle to effectively capture large-scale contextual information, resulting in shortcomings when extracting detailed cultivation information across different growth stages. Compared to CNNs, Transformers can capture long-range dependencies in images through their self-attention mechanism, thereby enhancing target recognition capabilities in complex scenes. However, their global modelling process focuses more on semantic consistency, resulting in relatively weaker capabilities for depicting fine-grained boundaries and local high-frequency structures. Furthermore, Transformer methods typically rely on large-scale training data and have high computational complexity, making them difficult to adapt to the deployment requirements of UAV remote sensing image tasks. Compared to CNNs and Transformers, the Mamba model demonstrates greater application potential in UAV remote sensing tasks, achieving a good balance between efficiency and accuracy. Currently, research on Mamba in the field of agricultural computer vision is still in its exploratory phase, and its application in the precise extraction of information from remote-sensed crop cultivation areas remains relatively limited. There is significant room for research into the precise extraction of information regarding celery cultivation across multiple growth stages.

From a data perspective, most existing studies rely on medium- to low-resolution satellite imagery, which struggles to meet the demand for detailed monitoring at the field scale. Furthermore, previous work has primarily focused on major crops such as rice and wheat, while research on fine-textured vegetable crops like celery remains relatively scarce. Additionally, these studies have not performed detailed segmentation of crops across different growth stages, limiting the models’ feature extraction capabilities in tasks involving detailed extraction across multiple growth stages.

In terms of practical agricultural remote sensing applications, celery is often planted in stages and intermixed with other crops in complex open-field environments. Plants at different growth stages exhibit high similarity in spectral characteristics and spatial morphology. This, combined with spectral confusion and interference among multiple crops in mixed plantings, further increases the difficulty of extraction. This places higher demands on the model’s ability to capture subtle differences such as canopy texture and boundary morphology. At the same time, interference from shadow occlusion and bare soil backgrounds further compounds the challenges of fine-grained extraction tasks. Therefore, there is an urgent need to explore more effective model optimization strategies to fill the gap in fine-grained crop remote sensing research across multiple growth stages.

To address these challenges, this paper proposes WaveletUMamba, a wavelet-enhanced state-space model, for the precise extraction of celery cultivation areas across multiple growth stages in UAV remote sensing imagery. While retaining Mamba’s efficient global modeling capabilities, this method effectively enhances the representation of local high-frequency details and improves the discrimination accuracy between different growth stages by introducing a global semantic guidance mechanism.

## 3 Study area and data

**Figure 1:**
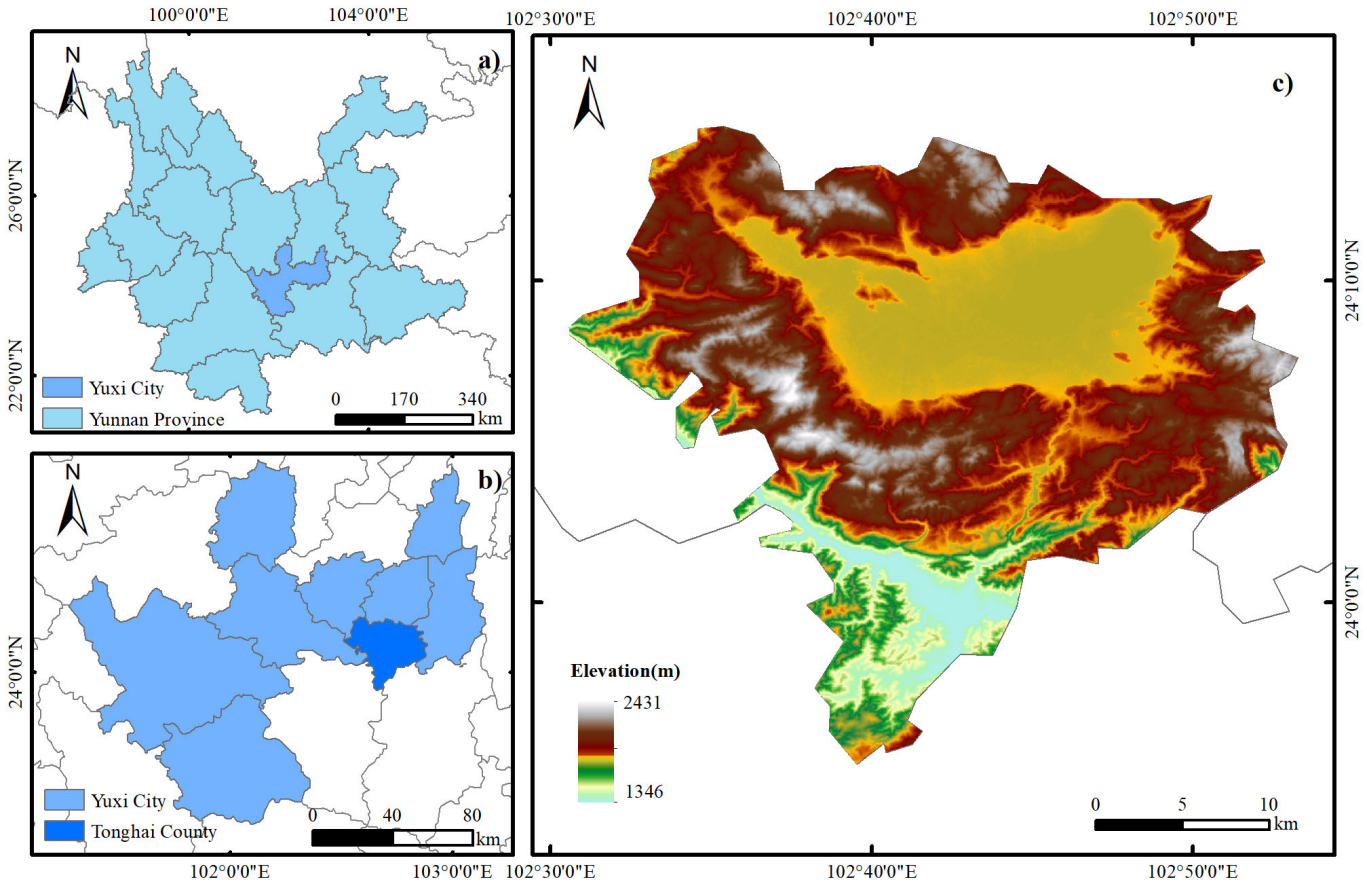
Geographical location of the study area. **(a)** Yunnan Province, **(b)** Yuxi City, and **(c)** Tonghai County.

The data collection area is located in Dashu Community, Xiushan Subdistrict, Tonghai County, Yuxi City, Yunnan Province, China (102°45′E, 24°7′N), as shown in **Figure 1**. Tonghai County is situated in the central part of the Yunnan Plateau, with an average elevation of approximately 1,820 meters, an average annual temperature of about 16.3 °C, and annual precipitation of approximately 903.7 millimeters. With its mild climate and abundant sunlight, this region is one of the major open-field vegetable production bases in southwest China. This vegetable-growing area features a typical highland open-field mixed cropping pattern characterized by “small plots and multiple varieties,” with widespread cultivation of various vegetable crops such as celery, lettuce, cilantro, cabbage, and Chinese cabbage. The total area under vegetable cultivation exceeds 297,300 mu.

The planting area adopts a phased sowing and crop rotation strategy, with different crops spatially interspersed. High similarity in spectral responses, canopy structures, and textural characteristics exists both within and across crop types, posing significant challenges for the fine-grained interpretation and classification of high-resolution remote sensing imagery while providing a representative experimental setting for this study.

The celery variety studied in this research is Chinese celery. This paper classifies phenological stages based on the standard for the nutritional growth period of celery published by China Agricultural Press on the Agricultural Professional Knowledge Service Platform. Given that the study area employs a transplanted cultivation model, the germination stage is not within the scope of this study’s observations. Therefore, the growth process after transplanting is divided into four key stages. The agronomic definitions of each stage and their corresponding drone imagery features are as follows:

1. Seedling stage: Refers to the period from cotyledon expansion to the full expansion of 4–5 true leaves. During this period, plants are short and have sparse canopies. Drone imagery shows a high proportion of exposed soil in the background, with plants distributed sporadically as isolated spots.
2. Early Leaf Development Stage: This refers to the period from 4–5 true leaves to 8–9 true leaves. Plants enter the early vegetative growth phase, and the canopy begins to expand laterally. However, from an aerial perspective, distinct soil gaps between adjacent plants are still visible, and leaf texture is relatively smooth.
3. Rapid Leaf Development Stage: This refers to the stage from 8–9 true leaves to 11–12 true leaves. During this period, vegetative growth is vigorous, and petioles thicken rapidly. The canopy expands rapidly and overlaps, leaves appear bright green with high reflectance, and gaps between plants have largely disappeared.
4. Harvesting Stage: This refers to the period from the end of the peak clump growth stage until the plants meet maturity criteria. At this point, the canopy is fully closed, and its spatial morphology tends toward stability. As the leaves age, some leaf margins exhibit slight yellowing or darkening, and the high-frequency wrinkles and shadow patterns on the canopy surface become significantly more pronounced.

**Figure 2:**
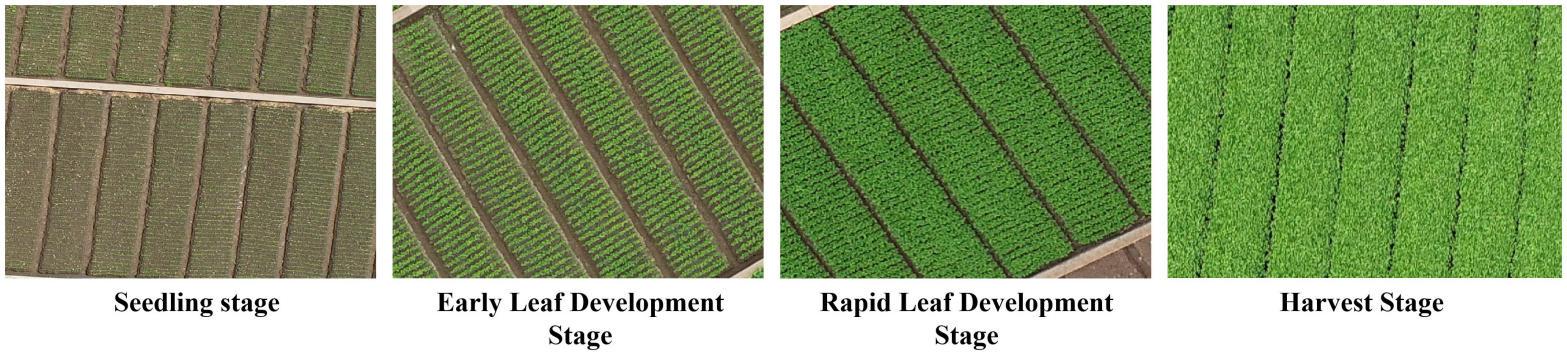
Imagery of celery at different growth stages.

To capture different seasonal conditions, enhance data diversity, and ensure a balanced sample across growth stages, this study conducted three rounds of drone data collection on May 1, July 27, and November 23, 2025. The imagery acquired during each aerial survey fully covered the four typical phenological stages of celery (as shown in **Figure 2**).

The data acquisition platform utilized a DJI Phantom 4 multirotor drone equipped with a high-resolution RGB camera with 20 million effective pixels. To minimize the impact of changes in shadow orientation and the sun’s altitude on the radiometric characteristics of the images, all aerial photography missions were strictly conducted between 9:00 a.m. and 11:00 a.m. under cloudless or partly cloudy skies with light winds. The drone operated in orthorectification mode at a flight altitude of 50 meters, and detailed flight parameters are shown in **Table 2**.

**Table 2:** UAV flight parameters.

Parameter	Value
Flight altitude	50 m
Forward overlap	80%
Side overlap	70%
Flight speed	3m/s
Number of GCPs	6
Image resolution	5472 × 3648

After quality screening of the raw aerial imagery—excluding images with distortion, blurring, and strong reflections—a total of 2,700 valid images were retained. To adapt to the input dimensions of the deep learning model and enhance the representation of local features, this study employed a sliding window method to crop the raw imagery into 1,024 × 1,024-pixel image tiles. This tile size effectively balances contextual information with detail resolution: it fully covers celery planting beds and adjacent plots while retaining sufficient spatial context, avoids introducing excessive irrelevant crops due to overly large tiles, and preserves local details such as leaf texture. Since the study area employs an open-field mixed cropping system, the images naturally contain a large number of background pixels, including bare soil, field ridges, and non-target crops. To avoid extreme class imbalance and reduce unnecessary computations, this study excluded tiles containing only background or non-target areas, ultimately constructing an image dataset comprising 4,039 valid samples.

**Figure 3:**
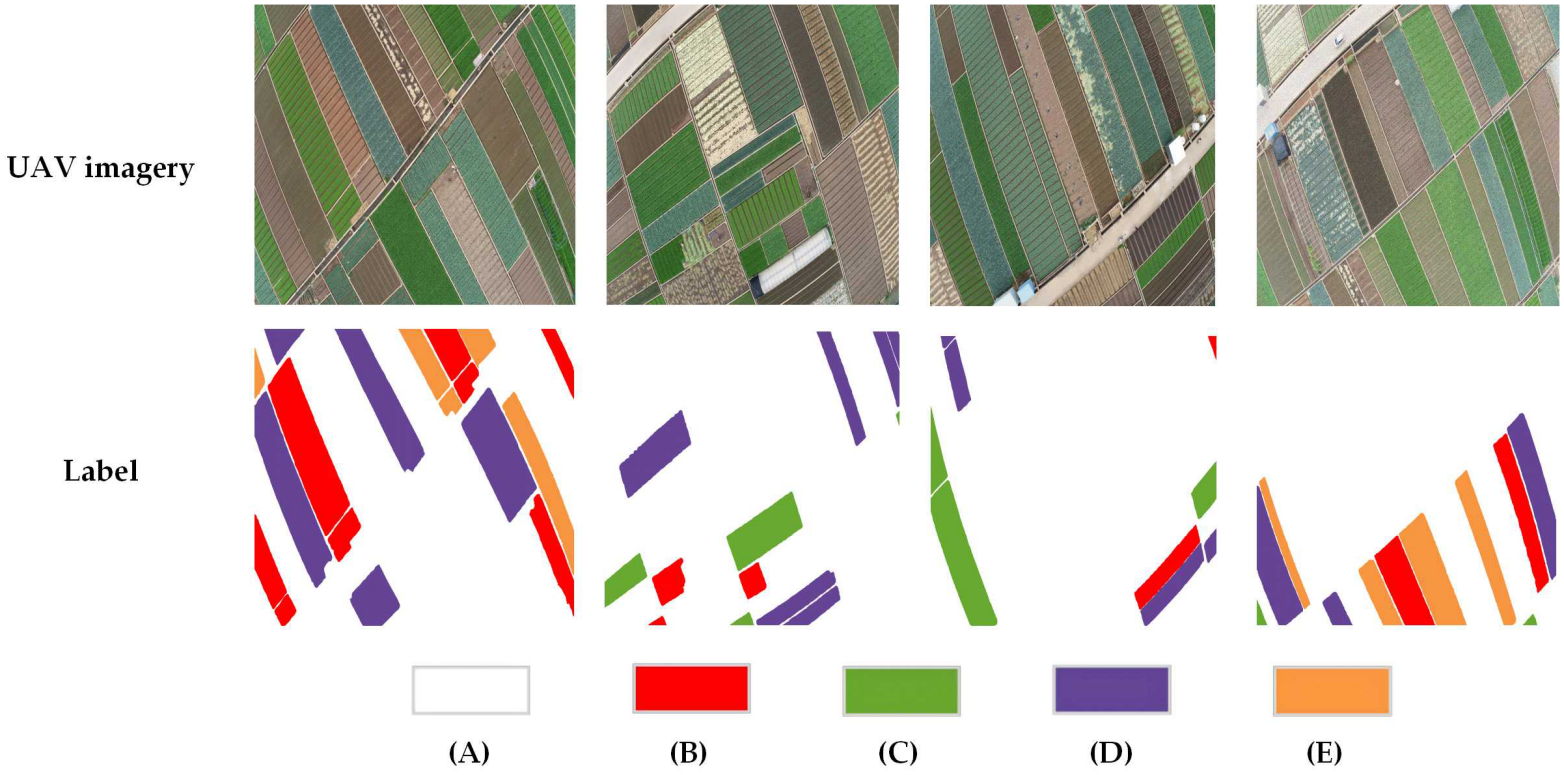
Examples of pixel-level annotation for UAV imagery: **(A)** Background; **(B)** Harvest stage; **(C)** rapid leaf development stage; **(D)** Seedling stage; **(E)** early leaf development stage.

During the sample annotation phase, a pixel-level manual annotation method was employed, using the LabelMe tool to annotate 4,039 image slices one by one. Based on the previously established criteria for determining celery growth stages, target areas were annotated by drawing polygonal contours, as illustrated in **Figure 3**. In accordance with the research objectives, all pixels were classified into five semantic categories: background, celery seedling stage, early leaf development stage, rapid leaf development stage, and harvesting stage.

To improve the consistency and reliability of the annotation results, quality control for all samples was conducted using a combination of independent annotation by two annotators and cross-checking. For areas where different growth stages overlap and for samples with ambiguous classifications, manual corrections were performed using high-resolution ground-level photographs collected in the field to ensure the accuracy of class classification and the precision of boundary annotations. To quantitatively assess annotation consistency, this study further randomly selected 100 sample images from the dataset. Two annotators independently annotated these images, and Cohen’s Kappa coefficient was calculated based on pixel labels. The results showed that the Kappa coefficient between the two annotators reached 0.87, indicating that the dataset possesses a high degree of consistency and reliability.

Furthermore, to quantitatively analyze the sample distribution characteristics and category balance of the dataset, this study conducted pixel-level statistics on the semantic labels of the annotated dataset. The results of the pixel proportions for each category are shown in **Table 3**. Among them, the background category accounted for 70.98% of the pixels, primarily corresponding to bare soil, field ridges, roads, weeds, and non-target crop areas in open-field agricultural scenes. The combined proportion of target pixels across the four growth stages of celery was 29.02%, indicating an imbalance between the background and target categories. This distribution is not due to biases in data collection or annotation but rather reflects an inevitable characteristic of open-field mixed-cropping environments. In response to this distribution characteristic, this study removed redundant image tiles containing only background features and no target crops during the preprocessing stage. Additionally, feature space diversity for the target class was enhanced during training by introducing strategies such as random scale scaling and random 90° rotations.

**Table 3:** Distribution of sample pixels.

Category	Pixel ratio(%)
Background	70.98
Seedling stage	8.36
Early leaf development stage	5.09
Rapid leaf development stage	6.95
Harvest stage	8.61

Finally, the dataset was split into a training set (2,827 images), a validation set (807 images), and a test set (405 images) in a 7:2:1 ratio, with no spatial overlap between the subsets.

## 4 Research methods

**Figure 4:**
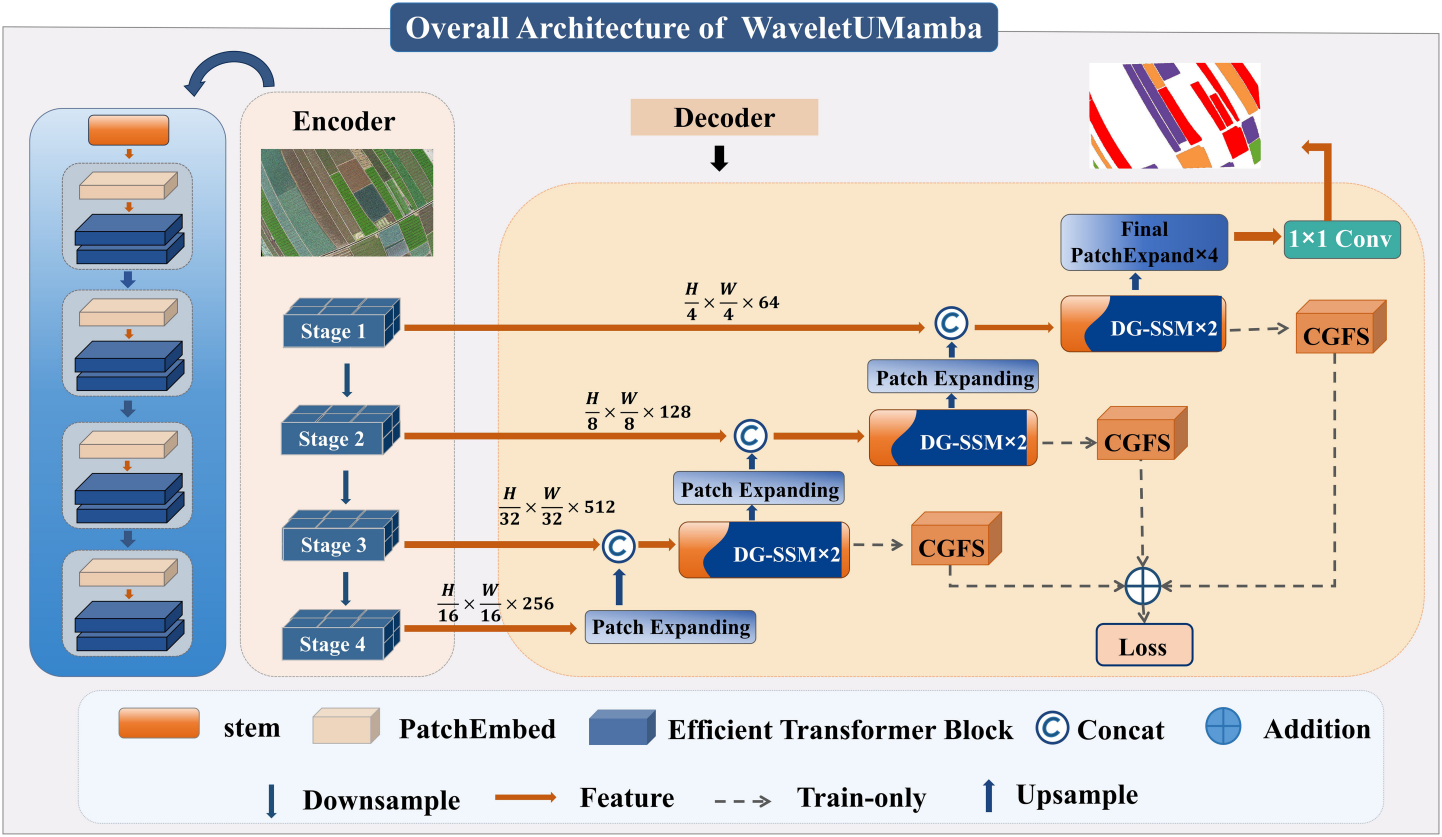
Overall architecture of WaveletUMamba.

The overall framework of WaveletUMamba is based on the classic encoder–decoder architecture of U-Net (as shown in **Figure 4**). The encoder uses ResT (Zhang and Yang, 2021) as its backbone network to extract multiscale hierarchical semantic features. The decoder consists of two core modules: DG-SSM and CGFS.

Among these, the DG-SSM module uses the VSSBlock (Liu et al., 2024) from the Mamba architecture as its core semantic modeling unit to extract global semantic features. To address the insufficient response to fine details in pure semantic state-space modeling, the LFM-HFE submodule was further designed within the DG-SSM module. It performs a frequency-domain decomposition of features using discrete wavelet transform, extracts high-frequency components, and reconstructs and enhances them to strengthen information regarding edges, textures, and fine-grained structures. In terms of the fusion mechanism, DG-SSM performs collaborative modeling of the global semantic features provided by Mamba and the high-frequency detail features provided by LFM-HFE. It then filters and recalibrates the fusion results using spatial and channel attention, achieving adaptive complementarity between global semantic information and local detail information, thereby enhancing the model’s ability to represent fine-grained objects and transitional regions during growth.

CGFS serves as an auxiliary supervision module, providing context-guided and deep supervision constraints on the fused features during the decoding process. While ensuring local responsiveness, it introduces global semantic constraints to improve the consistent representation of features across different scales, thereby further alleviating semantic confusion between different growth stages.

### 4.1 Low-frequency masked high-frequency enhancer

**Figure 5:**
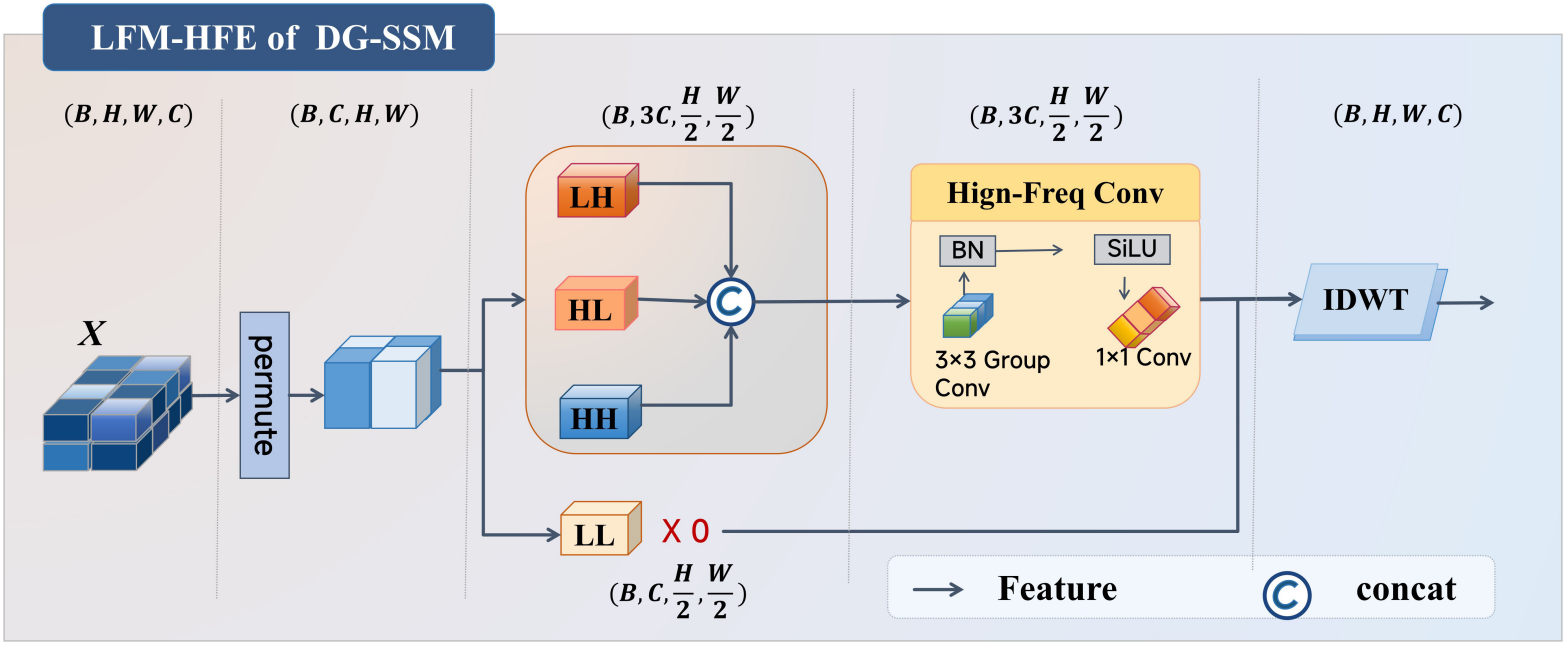
Architecture of the LFM-HFE module.

Although the existing Mamba architecture demonstrates the advantage of linear computational complexity in terms of global receptive field modeling and computational efficiency, its selective scanning mechanism is inherently biased toward the propagation of low-frequency information. As a result, local high-frequency features, such as leaf boundaries and inter-plant gaps, are prone to smoothing and information loss, leading to insufficient sensitivity to fine-grained textures. To address this limitation, the proposed Low-Frequency Masked High-Frequency Enhancer (LFM-HFE) is designed, as illustrated in **Figure 5**. Specifically, the Haar discrete wavelet transform (Zhang, 2019) is employed to decompose feature representations in the frequency domain, enabling the explicit extraction and reconstruction of high-frequency components to enhance boundary, texture, and fine-grained structural information.

First, given the input feature map $\text{X}\in {\text{R}}^{\text{H}\times \text{W}\times \text{C}}$, spatial resampling is performed by partitioning adjacent pixels into four sub-regions, as formulated in Equation 1.

(1) $$\{\begin{array}{c} x_{1}=\frac{X\times (2i,2j)}{2}\\ x_{2}=\frac{X\times (2i+1,2j)}{2}\\ x_{3}=\frac{X\times (2i,2j+1)}{2}\\ x_{4}=\frac{X\times (2i+1,2j+1)}{2} \end{array}$$

Based on the above local neighborhood partitioning, the wavelet decomposition process is described in Equation 2.

(2) $$\{\begin{array}{c} X_{ll}(i,j)=x_{1}+x_{2}+x_{3}+x_{4}\\ X_{lh}(i,j)=-x_{1}-x_{3}+x_{2}+x_{4}\\ X_{hl}(i,j)=-x_{1}+x_{3}-x_{2}+x_{4}\\ X_{hh}(i,j)=x_{1}-x_{3}-x_{2}+x_{4} \end{array}$$

where X_ll_ denotes the low-frequency component, and X_lh_, X_hl_, and X_hh_ represent the high-frequency detail components in the horizontal, vertical, and diagonal directions, respectively. To enhance the model’s responsiveness to boundary and texture structures, only the high-frequency components are further modeled in this work, while the reconstruction contribution of the low-frequency component is discarded by setting (X_ll_ = 0). This design encourages the network to focus on optimizing high-frequency details along the horizontal, vertical, and diagonal directions.

Subsequently, depthwise separable convolution followed by pointwise convolution is applied to the high-frequency coefficients for local detail modeling. A two-dimensional inverse Haar discrete wavelet transform is then employed to restore the spatial resolution, thereby generating high-frequency compensation features enriched with edge and texture information. These features provide more refined local details for subsequent fusion with the state-space branch. The overall process is described in Equations 3–5.

(3) $$X_{ll}=0$$

(4) $$X_{high}=Concat(X_{lh},X_{hl},X_{hh})$$

(5) $${\hat{X}}_{high}=\phi \left(X_{high}\right)$$

where *ϕ*(·) denotes a nonlinear transformation composed of depthwise separable convolution and pointwise convolution. During the reconstruction stage, the enhanced high-frequency components are mapped back to the spatial domain using the inverse discrete wavelet transform. The reconstruction process is formulated in Equation 6.

(6) $$\{\begin{array}{c} x_{1}=\frac{X_{ll}-X_{lh}-X_{hl}+X_{hh}}{2}\\ x_{2}=\frac{X_{ll}-X_{lh}+X_{hl}-X_{hh}}{2}\\ x_{3}=\frac{X_{ll}+X_{lh}-X_{hl}-X_{hh}}{2}\\ x_{4}=\frac{X_{ll}+X_{lh}+X_{hl}+X_{hh}}{2} \end{array}$$

The final output feature $\hat{X}\in R^{H\times W\times C}$ is obtained by filling the reconstructed results into their corresponding spatial locations.

Through the above frequency-domain decomposition-enhancement-reconstruction mechanism, the model is able to effectively model high-frequency structural responses, thereby improving its capability to perceive boundary details and local texture variations, while providing more discriminative feature representations for subsequent state-space modeling.

### 4.2 Dual-gated frequency–spatial state space module

**Figure 6:**
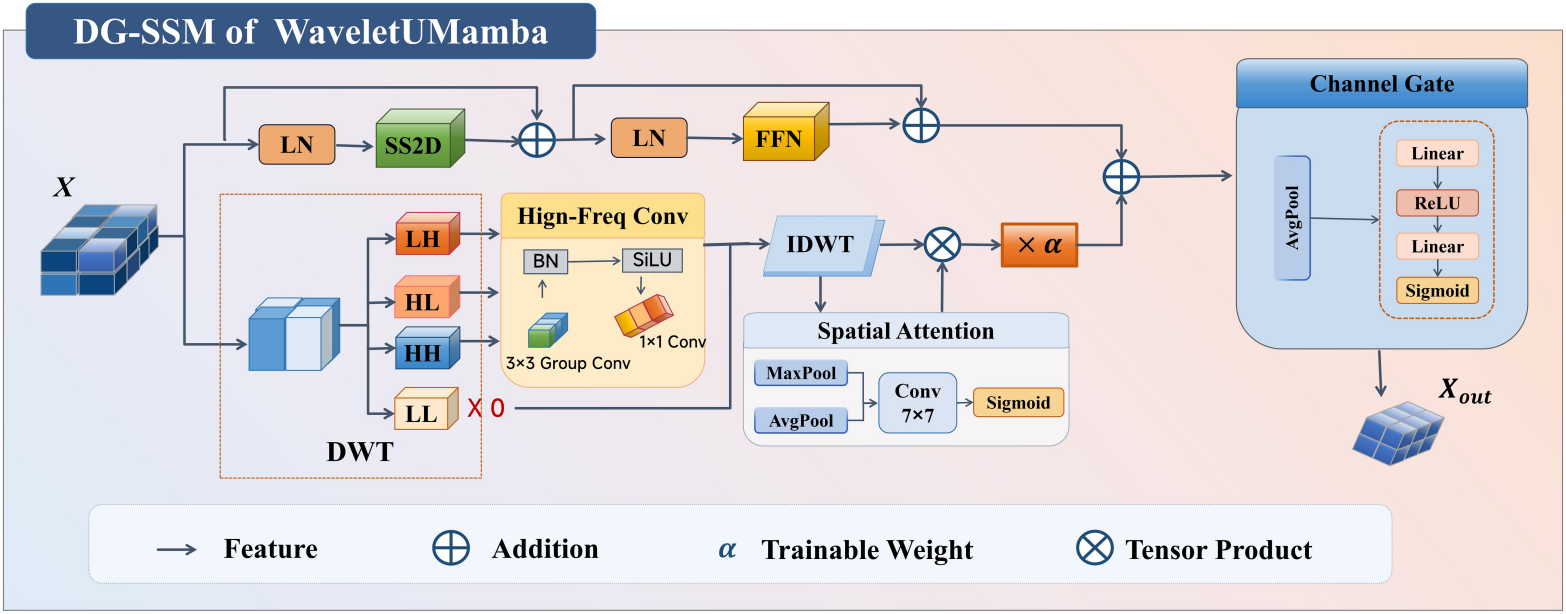
Architecture of the DG-SSM module.

The DG-SSM Block is the core module in the decoder and is responsible for feature reconstruction. It adopts a dual-branch parallel architecture to fuse the local detail features extracted by the LFM-HFE with the global semantic features extracted by the Mamba backbone. A dual-gated mechanism operating in both the spatial and channel dimensions is further introduced for feature selection and recalibration, as illustrated in **Figure 6**.

First, the main branch employs the VSSBlock to extract the global feature X_main_. The VSSBlock is a Mamba-based state-space modeling function that captures long-range spatial dependencies and generates global semantic representations, as formulated in Equation 7.

(7) $$X_{\text{main}}=VSSBlock(X)$$

Meanwhile, the auxiliary branch receives the high-frequency detail feature X_detail_ extracted by the LFM-HFE. To suppress redundant high-frequency responses, a spatial attention mechanism is introduced to adaptively select informative high-frequency features. Specifically, max pooling and average pooling are performed along the channel dimension, followed by a 7×7 convolution to generate the spatial mask, as described in Equations 8, 9.

(8) $$S_{mask}=\sigma (Conv_{7\times 7}([MaxPool(X_{detail}),AvgPool(X_{detail})]))$$

(9) $$X_{detail}^{\prime }=X_{detail}⊗S_{mask}$$

where σ denotes the Sigmoid activation function, and ⊗ represents element-wise multiplication.

Subsequently, the global semantic feature and the filtered high-frequency detail feature are fused through a residual connection. Here, α is a learnable scalar parameter that adaptively controls the injection strength of the high-frequency information, as formulated in Equation 10.

(10) $$X_{fused}=X_{main}+\alpha X_{detail}^{\prime }$$

Finally, the fused features are recalibrated in the channel dimension using a channel attention mechanism as a gating function, followed by Layer Normalization to produce the final output feature *X_out_*, as shown in Equation 11.

(11) $$X_{out}=LN(SE(X_{fused})⊗X_{fused})$$

### 4.3 Context-guided feature supervision module

**Figure 7:**
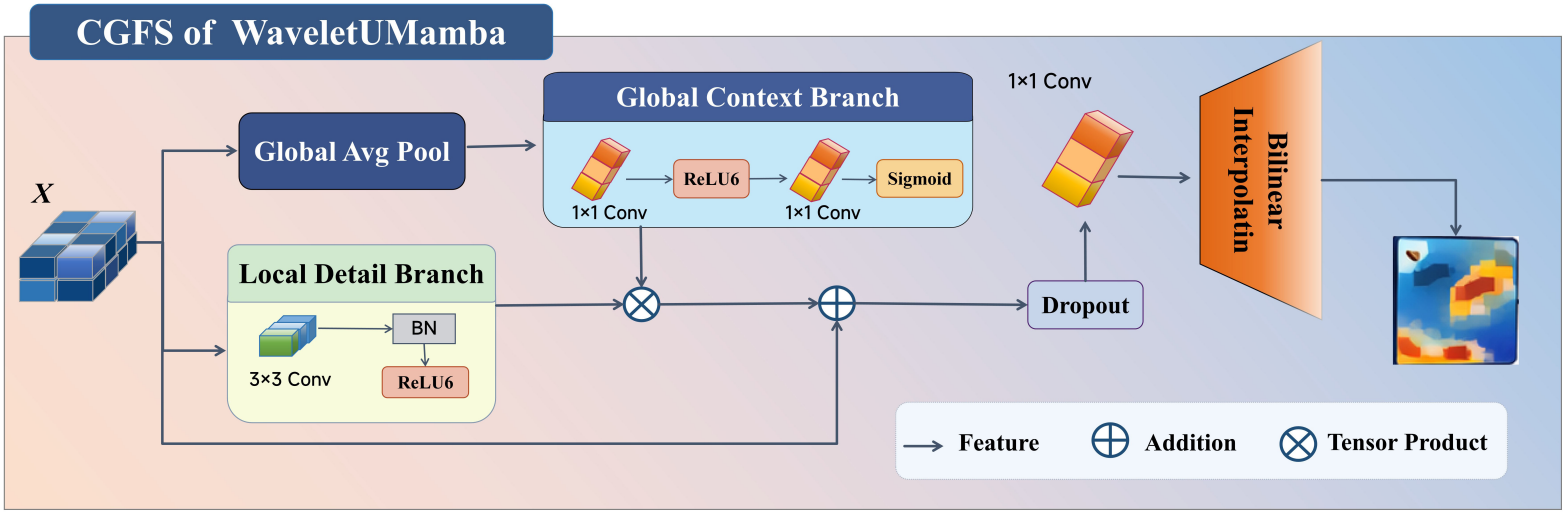
Architecture of the CGFS module.

To further enhance feature representation consistency and class discriminability during the decoding stage, a CGFS module is proposed, as illustrated in **Figure 7**. By integrating local detail modeling with a global context modulation mechanism, the CGFS module adaptively enhances decoder features and serves as an auxiliary supervision branch to guide multi-scale feature learning.

During training, the CGFS module is embedded into each intermediate layer of the decoder. It extracts local texture information from the fused features of each decoding stage, guides feature learning using global context weighting, and computes an auxiliary loss. During inference, the CGFS module is removed and introduces no additional computational overhead.

Assume that the input feature of the i decoder stage is $\text{F}\in {\text{R}}^{{\text{H}}_{\text{i}}\times {\text{W}}_{\text{i}}\times {\text{C}}_{\text{i}}}$. The CGFS module first employs depthwise separable convolution to extract the local texture feature F_local_, as formulated in Equation 12.

(12) $$F_{local}=ReLU6(BN(DWConv_{3\times 3}(F)))$$

Next, global contextual guidance is obtained by applying global average pooling to the input feature, followed by two pointwise convolutions to generate the channel attention weight $W_{ctx}\in R^{1\times 1\times C_{i}}$, as shown in Equation 13.

(13) $$W_{ctx}=\sigma (W_{2}(ReLU6(W_{1}(AdaptiveAvgPool(F)))))$$

where W_1_ and W_2_ denote the 1×1 convolution operations for channel reduction and channel expansion, respectively. Subsequently, the contextual weight is used to dynamically reweight the local texture feature, which is then fused with the original feature through a residual connection, as described in Equation 14.

(14) $$F_{guided}=(F_{local}⊗W_{ctx})+F$$

where ⊗ denotes element-wise multiplication.

Finally, the fused feature is regularized by a Dropout layer, projected to the target class space through a 1×1 convolution, and upsampled to the original image resolution *H*×*W* using bilinear interpolation to generate the auxiliary prediction ${\hat{Y}}_{aux}$, as formulated in Equation 15.

(15) $${\hat{Y}}_{aux}=UpSample(Conv_{1\times 1}(Dropout(F_{guided})))$$

During training, the CGFS module serves as a deep supervision branch for loss computation, thereby enhancing the semantic consistency of intermediate-layer features and improving the discriminative stability of the model across multi-scale feature representations.

### 4.4 Evaluation metrics

Since the core task of this study is pixel-level semantic classification of high-resolution remote sensing imagery, a comprehensive evaluation framework was established to objectively assess the performance of the proposed WaveletUMamba model for multi-growth-stage celery planting area extraction. The evaluation framework consists of two aspects: pixel-level classification performance and model complexity (Wang et al., 2020).

For segmentation evaluation, Intersection over Union (IoU) and mean Intersection over Union (mIoU) are adopted as primary metrics to quantify the overlap between predicted results and ground truth annotations. Specifically, IoU provides a strict measure of how well the model captures irregular boundaries and spatial structures of celery across different growth stages, while mIoU reflects the overall classification consistency across all categories, as described in Equations 16, 17.

(16) $$IoU=\frac{TP}{TP+FP+FN}$$

(17) $$mIoU=\frac{1}{N}\sum_{i=1}^{N}IoU_{i}$$

Here, true positives (TP) denote the number of correctly predicted positive pixels, false positives (FP) represent negative pixels incorrectly classified as positive, true negatives (TN) correspond to correctly predicted negative pixels, and false negatives (FN) indicate positive pixels incorrectly classified as negative.

In addition, to further evaluate pixel-level classification performance, Precision, Recall, and F1-score are introduced as complementary metrics, enabling a more detailed analysis of the model’s ability to distinguish between adjacent growth stages. Considering that the dominance of background classes may bias the Overall Accuracy (OA), this study jointly employs OA and the Kappa coefficient to provide a more reliable and statistically robust evaluation, as described in Equations 18–22.

(18) $$Precision=\frac{TP}{TP+FP}$$

(19) $$Recall=\frac{TP}{TP+FN}$$

(20) $$F1=2\times \frac{Precision\times Recall}{Precision+Recall}=\frac{2\times TP}{2\times TP+FP+FN}$$

(21) $$OA=\frac{\sum_{i=1}^{N}p_{ii}}{\sum_{i=1}^{N}\sum_{j=1}^{N}p_{ij}}$$

(22) $$Kappa=\frac{OA-p_{e}}{1-p_{e}},\;p_{e}=\frac{\sum_{i=1}^{N}\left(\sum_{j=1}^{N}p_{ij}\times \sum_{j=1}^{N}p_{ji}\right)}{T^{2}}$$

In these formulations, *N* denotes the total number of classes, *p_ij_* represents the number of pixels whose ground truth class is *i* but are predicted as class *j*, and *p_ii_* corresponds to the correctly classified pixels of class *i*. The total number of pixels is given by $T=\sum_{i=1}^{N}\times \sum_{j=1}^{N}p_{ij}$, and *p_e_* denotes the expected agreement by chance.

Considering the lightweight requirements of UAV-based agricultural remote sensing applications, the number of parameters (Params) and the number of floating-point operations (GFLOPs) were further adopted to quantify the model size and computational cost. By jointly analyzing the accuracy and complexity metrics, the proposed model was evaluated to determine whether it can improve extraction performance while maintaining computational efficiency and practical deployability.

## 5 Results and analysis

### 5.1 Experimental setup

To ensure the reliability and reproducibility of the experimental results, all experiments were conducted under a unified hardware and software environment. Specifically, the operating system was Ubuntu 20.04.6 LTS, the deep learning framework was PyTorch 2.5.1, the GPU was an NVIDIA RTX A4000 with 16 GB of memory, the CPU was an Intel Xeon Silver 4208, Python 3.8 was used as the programming language, and CUDA 11.8 was employed for accelerated computation.

All models followed the same data preprocessing pipeline and training protocol. The input image resolution was set to 1024×1024. During training, a consistent data augmentation strategy was adopted, including random scaling with a scale factor ranging from 0.5 to 1.5 and random 90° rotations. During the validation and testing stages, only normalization was applied, without any random data augmentation.

During training, the AdamW optimizer was used for parameter optimization, with an initial learning rate of 6×10^−4^ and a weight decay of 2.5×10^−4^. The learning rate was scheduled using the Cosine Annealing Warm Restarts strategy, where the initial restart period T_0_ was set to 15 and the period multiplication factor T_mult_ was set to 2. The batch size was set to 8, and all models were trained for 200 epochs. The Cross-Entropy Loss was adopted to supervise pixel-wise classification. To ensure reproducibility, the random seed was fixed to 42, and the random number generators of Python, NumPy, and PyTorch were initialized consistently.

All comparison models were reproduced based on their officially released implementations. Except for the network architectures and their default initialization schemes, all models were trained and evaluated using the same dataset split, input resolution, data augmentation strategy, training epochs, optimizer settings, and evaluation metrics under the same hardware and software environment, thereby ensuring the fairness and reproducibility of the experimental results.

### 5.2 Comprehensive performance comparison

Systematic comparative experiments were conducted on the constructed high-resolution UAV multi-growth-stage celery dataset to comprehensively evaluate the effectiveness and generalization capability of the proposed method. Sixteen representative state-of-the-art semantic segmentation models were selected as baseline methods, covering the three mainstream architectural paradigms: CNN, Transformer, and Mamba. The overall performance comparison is presented in **Table 4**. The results demonstrate that the proposed WaveletUMamba achieves superior performance across multiple key evaluation metrics, providing a favorable balance between extraction accuracy and computational efficiency.

**Table 4:** Quantitative comparison of models across three mainstream architectural paradigms.

Method	Model	Params (M)	GFLOPs (G)	OA	mIoU	mF1	Kappa
CNN	UNet (Ronneberger et al., 2015)	31.04	771.97	94.38	78.28	87.47	88.57
	SegNet (Badrinarayanan et al., 2017)	62.48	289.48	93.00	75.00	85.31	85.63
	DeConvNet (Noh et al., 2015)	251.80	829.96	85.24	55.82	70.17	69.23
	DeepLabV1 (Chen et al., 2014)	42.51	720.44	95.53	82.10	89.95	90.86
	DeepLabV2 (Chen et al., 2017)	42.87	726.40	90.45	67.11	79.56	79.79
	DeepLabV3 (Chen et al., 2018b)	58.04	970.13	95.56	82.48	90.16	90.94
Transformer	BANet (Tsai et al., 2022)	12.71	85.16	96.82	86.56	92.66	93.48
	MANet (He et al., 2022)	35.86	216.51	97.15	87.52	93.20	94.14
	SwinUNet (Cao et al., 2023)	41.39	189.50	96.14	84.53	91.41	92.10
	DCSWin (Wang et al., 2022a)	45.63	190.03	96.57	85.44	91.96	92.97
	UNetFormer (Wang et al., 2022b)	**11.72**	**47.03**	96.88	86.50	92.61	93.57
Mamba	CM-Unet (Cui et al., 2023)	13.41	51.75	95.58	82.34	90.08	90.94
	EfficientPyramidMamba (Wang et al., 2025)	28.76	76.16	93.69	75.99	85.94	87.17
	MambaUnet (Wang et al., 2024)	13.88	51.63	95.49	82.92	90.49	90.83
	RS3-Mamba (Fang et al., 2025)	43.32	158.26	96.23	84.17	91.22	92.25
	UnetMamba (Zhu et al., 2024)	14.76	100.46	97.09	87.77	93.37	94.02
	WaveletUMamba	15.61	104.50	**97.39**	**88.70**	**93.90**	**94.65**

All performance metrics are reported as percentages (%). The best results are highlighted in bold.

As shown in **Table 4**, the overall performance of CNN-based models is relatively limited. Among them, DeepLabv3 performed best among CNN-based methods, achieving an mIoU of 82.48%. However, its computational overhead is significantly higher than that of other compared models, reaching as high as 970.13 GFLOPs. Although its larger receptive field enhances its scene understanding capabilities, the excessive computational complexity limits its practical deployment on resource-constrained agricultural drone platforms. Furthermore, some early CNN models perform noticeably poorly in complex agricultural field scenarios. For example, DeConvNet achieves an mIoU of only 55.82%, which is far lower than that of other mainstream models. This indicates that relying solely on local convolutional operations makes it difficult to adequately model the complex spatial relationships and long-range dependencies among crops, and that these models still face significant limitations in fine-grained class recognition and distinguishing complex backgrounds.

Compared to CNN architectures, Transformer models incorporating self-attention mechanisms achieved overall better feature extraction performance. Among them, MANet achieved an mIoU of 87.52%, making it the best model among Transformer-based methods, while SwinUNet—though the model with the lowest accuracy in this architecture, with an mIoU of 84.53%—still outperformed DeepLabv3, the best model in the CNN category. This indicates that Transformers can effectively capture long-range spatial dependencies through global feature modelling, which plays a positive role in distinguishing celery plots at different growth stages in complex farmland scenes. However, this performance improvement also comes at a significant computational cost. MANet’s parameter count and computational cost reach 35.86 M and 216.51 GFLOPs, respectively, while SwinUNet’s reach 41.39 M and 189.50 GFLOPs. This high resource consumption limits its application on edge devices to some extent.

In contrast, the Mamba architecture demonstrates a better balance between accuracy and efficiency. Experimental results show that most Mamba models can consistently achieve an mIoU of over 82% with relatively low computational overhead, approximately 50–160 GFLOPs. For example, UNetMamba has only 14.76M parameters, with a computational cost of 100.46 GFLOPs, and achieves an mIoU of 87.77%, outperforming both CNN- and Transformer-based methods in accuracy. By leveraging a selective scanning mechanism, this class of models achieves near-linear computational complexity and exhibits good scalability when processing high-resolution remote sensing imagery.

Among all the compared models, the WaveletUMamba proposed in this paper demonstrates the best overall performance. Its mIoU reaches 88.70%, with OA and Kappa metrics at 97.39% and 94.65%, respectively—all outperforming all comparison methods. Compared to DeepLabv3, the best-performing model among CNN architectures, WaveletUMamba achieves an approximately 6.22% increase in mIoU while significantly reducing the number of parameters and computational overhead. Compared to MANet, the best-performing model among Transformer architectures, our method further improves segmentation accuracy while reducing computational cost by approximately 51.7%. Compared to UNetMamba, the second-best model in the Mamba architecture, it achieves a 1.07% increase in mIoU while maintaining comparable computational efficiency.

**Figure 8:**
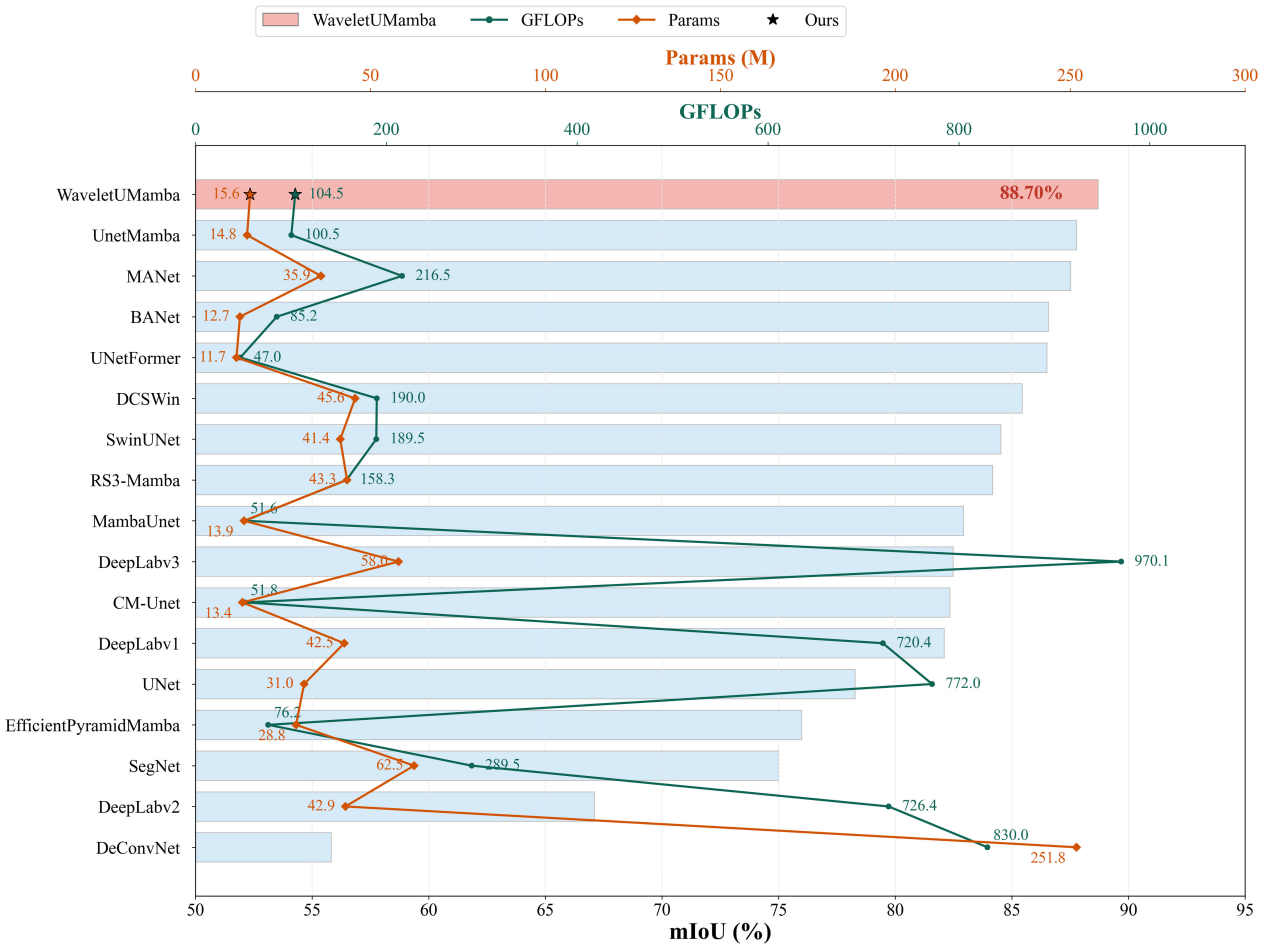
Performance comparison illustrating the trade-off between segmentation accuracy and computational efficiency.

**Figure 8** provides a comprehensive comparison of the various models across three dimensions: the number of parameters, computational complexity, and segmentation accuracy. It can be observed that CNN-based models generally suffer from high computational overhead with limited performance gains. While Transformer-based models improve extraction accuracy, their parameter size and computational cost remain high. In contrast, Mamba-based models are generally distributed in the lower-complexity region while maintaining high extraction accuracy, demonstrating a superior accuracy–efficiency trade-off. The WaveletUMamba model proposed in this paper achieved the highest mIoU with a relatively low number of parameters and computational complexity, positioning it at the forefront of the performance distribution. This indicates that it can effectively reduce model deployment costs while ensuring extraction accuracy, making it more suitable for resource-constrained agricultural drone remote sensing applications.

### 5.3 Qualitative analysis of representative models

To further analyze the extraction performance of different models across various growth stages of celery, this paper selected representative models with superior overall performance from three architectural categories—CNN, Transformer, and Mamba—and conducted a detailed comparative analysis with WaveletUMamba. **Table 5** presents the comparison results for Precision, Recall, F1, and IoU across different growth stage categories for each model.

**Table 5:** Quantitative comparison across different growth-stage categories for various models.

Growth stage category	Evaluation metric	DeepLabv3	MANet	RS3-Mamba	CM-Unet	UnetMamba	WaveletUMamba
Background	Precision	99.02	99.38	99.10	98.83	99.31	**99.47**
	Recall	97.99	**99.06**	98.86	98.28	98.86	99.03
	F1	98.50	99.22	98.98	98.55	99.09	**99.25**
	IoU	97.04	98.46	97.98	97.15	98.19	**98.51**
Seedling stage	Precision	88.31	91.10	90.04	88.02	90.41	**92.11**
	Recall	90.21	**94.89**	91.14	92.20	92.18	94.48
	F1	89.25	92.95	90.58	90.39	91.29	**93.28**
	IoU	80.59	86.83	82.79	82.47	83.97	**87.41**
Early leaf development stage	Precision	78.53	**87.99**	86.22	84.11	87.84	87.22
	Recall	88.37	87.69	85.74	83.71	89.64	**91.82**
	F1	83.16	87.84	85.98	83, 91	88.73	**89.46**
	IoU	71.17	78.32	75.41	72.28	79.74	**80.93**
Rapid leaf development stage	Precision	88.40	90.15	85.13	85.77	92.01	**92.62**
	Recall	86.87	91.52	91.35	86.09	**92.52**	91.10
	F1	87.63	90.83	88.13	85.93	**92.27**	91.85
	IoU	77.98	83.19	78.78	75.33	**85.64**	84.93
Harvest stage	Precision	91.88	**96.29**	94.62	92.01	95.37	95.89
	Recall	92.64	94.08	90.31	91.18	**95.55**	95.45
	F1	92.26	95.17	92.41	91.59	95.46	**95.67**
	IoU	85.63	90.78	85.90	84.49	91.31	**91.70**

All evaluation metrics are reported as percentages (%). The best results are highlighted in bold.

In terms of category performance, the early leaf development stage category has the lowest overall classification performance and is the most challenging to identify. At this stage, plants are in the early phase of foliage expansion. The canopy has not yet fully closed, and large areas of bare soil remain visible on the ground. Additionally, its morphological characteristics combine features from both the seedling stage and the rapid leaf development stage, and its spectral and textural features are similar to those of other categories, making it prone to confusion with adjacent growth stages. Most models achieved lower IoU values for this category compared to others. For example, DeepLabv3 achieved only 71.17% IoU, while the better-performing UNetMamba reached 79.74%. In contrast, WaveletUMamba further improved this figure to 80.93%, an increase of 1.19 percentage points over the second-best model, indicating that the proposed method can more effectively capture subtle feature differences in transitional regions between growth stages, thereby enhancing the ability to distinguish between easily confused classes.

In UAV imagery, celery seedlings are small during the seedling stage, with sparse canopies and a significant amount of bare soil in the background. Since the target area accounts for a small proportion of the image, it is easily affected by background noise and boundary uncertainty, thus placing higher demands on the model’s ability to perceive local details. Experimental results show that WaveletUMamba achieved an IoU of 87.41% for this class, outperforming all comparison models. Specifically, it outperformed MANet—the best-performing model among Transformer architectures—by 0.58 percentage points and UNetMamba by 3.44 percentage points.

For the rapid leaf development stage and harvest stage, since the plant canopy is largely closed, the target areas exhibit strong continuity and more stable class features, allowing all models to achieve high classification accuracy. Among them, WaveletUMamba achieved an IoU of 91.70% at the harvest stage, the best result among all models. In the rapid leaf development stage, it achieved an IoU of 84.93%, which was slightly lower than UNetMamba’s 85.64%. This suggests that while the frequency-domain enhancement mechanism improves recognition capabilities for challenging classes with weak inter-class distinctions, it may cause slight performance fluctuations in certain classes with relatively stable textural features. However, the overall impact is minimal.

Looking at the overall results, WaveletUMamba achieved superior classification performance across multiple growth stage categories, with the overall mIoU increasing to 88.70%. Compared to the second-best model, UNetMamba, the performance improvement was more significant for challenging categories such as the early leaf development stage and Seedling Stage, indicating that wavelet frequency-domain features and the state-space modeling mechanism can effectively address the original Mamba architecture’s inadequacy in representing local high-frequency information. Furthermore, by combining local detail modeling with a global context modulation mechanism, CGFS acts as an auxiliary supervision branch to guide multiscale feature learning. This enables the model to more accurately capture leaf textures, canopy boundaries, and transition zones between growth stages, thereby enhancing fine-grained classification capabilities in complex agricultural scenarios.

**Figure 9:**
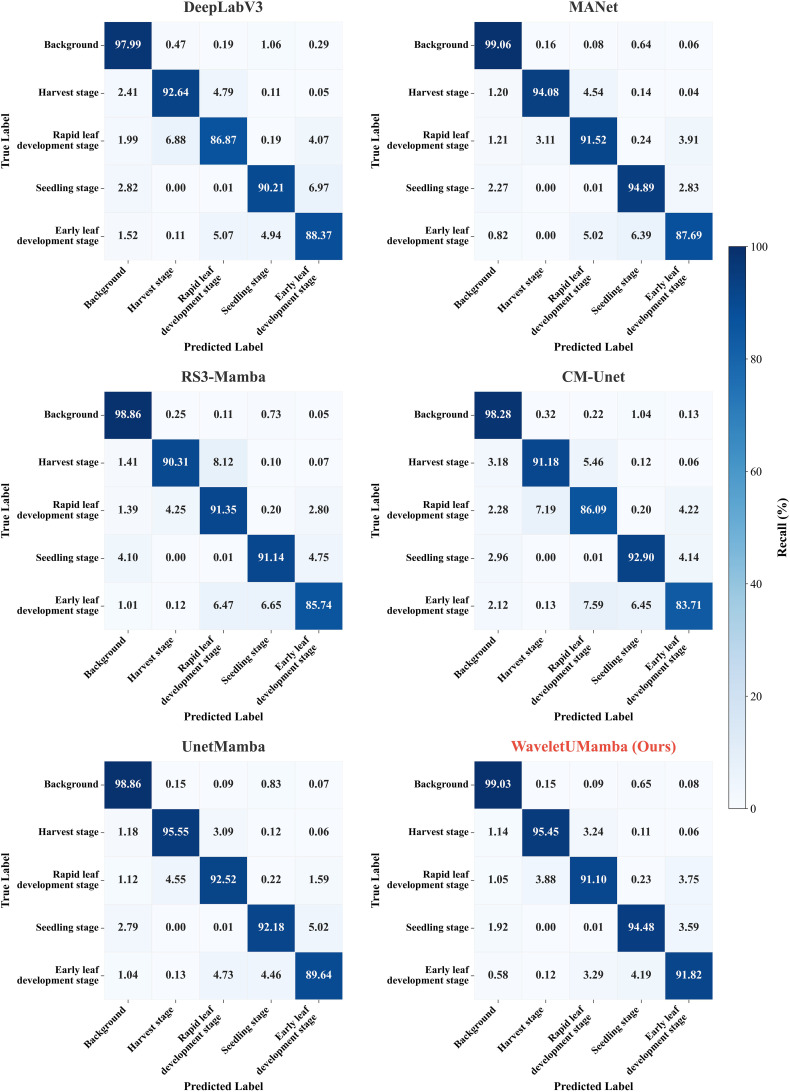
Row-normalized confusion matrices of representative models. The diagonal elements represent class-wise recall, while the off-diagonal elements indicate misclassification proportions between categories.

To further analyze the classification performance of each model across different growth stages, **Figure 9** shows the row-normalized confusion matrix for representative models of each architecture on the test set. The main diagonal represents the recall for each category, while the off-diagonal elements reflect the misclassification rates between different categories. By comparing the confusion distributions of different models, we can observe their varying performance in identifying adjacent growth stages.

The confusion matrix reveals that category confusion is primarily concentrated between adjacent growth stages. Among these, the early leaf development stage is the category most prone to misclassification. This stage marks a critical transitional period in growth, during which the plant canopy has not yet fully closed. The leaf morphology, coverage, and textural features of this stage are highly similar to those of adjacent growth stages, making classification confusion likely. For DeepLabv3, 5.07% and 4.94% of the pixels in the early leaf development stage were misclassified as rapid leaf development and seedling stages, respectively. UNetMamba’s misclassification rates for these categories were 4.73% and 4.46%, respectively. In contrast, WaveletUMamba reduced the misclassification rates for these two categories to 3.29% and 4.19%, respectively, while simultaneously increasing the recall rate for this category to 91.82%. These results indicate that the proposed method can more accurately capture subtle differences between different growth stages, effectively enhancing the ability to distinguish samples in transitional stages.

For the seedling stage, due to the small size of the plants and their scattered spatial distribution, there is a large amount of bare soil background surrounding the target area, and the boundary features are relatively blurred, making it more susceptible to interference from background noise. The confusion matrix reveals that the second-best model, UNetMamba, misclassified 5.02% of seedling-stage pixels as the early leaf development stage and another 2.79% as “Background.” In contrast, WaveletUMamba reduced the corresponding misclassification rates to 3.59% and 1.92%, respectively, while increasing the recall rate for this class to 94.48%.

Overall, WaveletUMamba demonstrates a higher proportion of the main diagonal and a lower distribution of off-diagonal errors across most categories, further validating that the proposed method can enhance the representation of local fine-grained features and global contextual information, effectively reducing category confusion between adjacent growth stages and thereby improving the fine-grained recognition capability of crop growth stages in complex agricultural scenarios.

**Figure 10:**
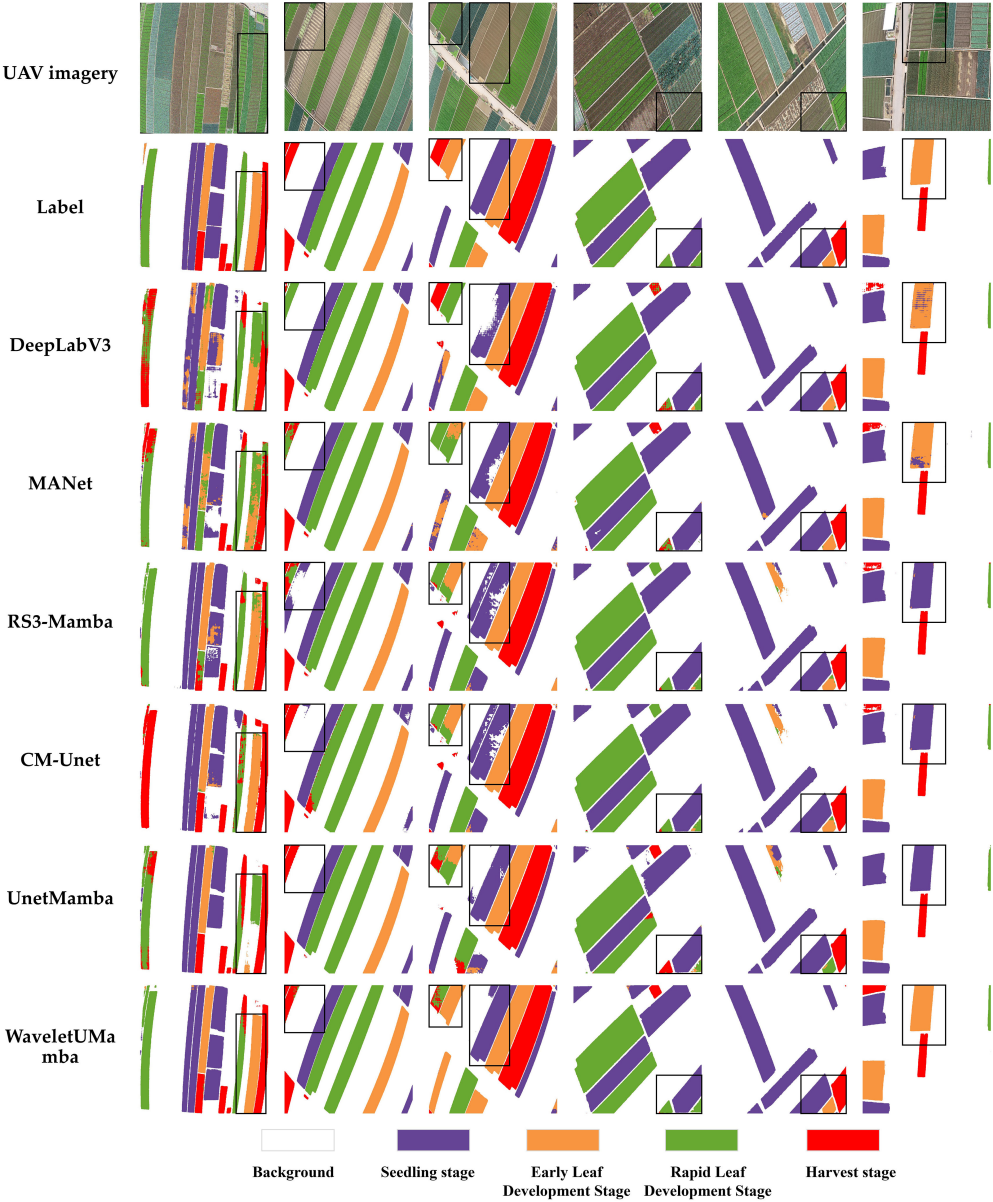
Visual comparison of segmentation results produced by representative models. The highlighted regions (black boxes) emphasize the differences in segmentation quality.

**Figure 10** presents a visual comparison of crop area extraction results from representative models applied to a high-resolution UAV-acquired celery dataset spanning different phenological stages. It is evident that DeepLabv3 is prone to internal fragmentation within areas in complex scenes. In the black-marked regions of the first and third columns, noticeable misclassified patches and local noise can be observed, leading to discontinuities in class boundaries within the same plot. MANet shows some improvement in area integrity and maintains good continuity for large plots. However, noticeable misclassified patches still exist, as shown in the black-boxed areas in the third and fourth columns.

In contrast, the state-space model-based RS3-Mamba, CM-Unet, and UNetMamba all further reduce the amount of internal noise and are able to better preserve the overall structure of the patches. In most scenarios, models based on the Mamba architecture are better at restoring the regular contours of patches, but some category adhesion still occurs at the boundaries between adjacent growth stages. For example, in the black-boxed areas of the third column, the boundaries of some fragmented plots show varying degrees of blending, resulting in deviations between the demarcation points between adjacent growth stages and the ground truth labels. This indicates that while the original Mamba architecture possesses strong global dependency modeling capabilities, there is still room for improvement in utilizing local high-frequency information in boundary regions.

Compared to all other models, WaveletUMamba produces segmentation results closest to the ground truth labels. In multiple black-box-marked regions, it not only effectively suppresses misclassification noise within the regions but also preserves the boundary structures of narrow, elongated patches, fragmented boundaries, and adjacent growth stages relatively completely. Particularly in the complex regions of the first and third columns, the model accurately restores patch contours and reduces category confusion between different growth stages, resulting in higher consistency between the predicted results and the ground truth labels.

### 5.4 Ablation study

To systematically evaluate the impact of each key component on model performance, this paper conducted a stepwise ablation analysis of the baseline model, LFM-HFE, DG-SSM, and CGFS modules under a unified experimental setup. The experimental results are shown in **Table 6**.

**Table 6:** Ablation study results of different model components.

LFM-HFE	DG-SSM	CGFS	Param(M)	FLOPS(G)	mIou	mF1	OA	Kappa
×	×	×	14.76	100.46	87.77	93.37	97.09	94.02
✓	×	×	16.38	104.47	87.81	93.40	97.10	94.05
✓	✓	×	16.42	104.50	87.90	93.45	97.11	94.10
×	×	✓	**13.94**	**100.45**	88.41	93.76	97.12	94.24
✓	✓	✓	15.61	104.50	**88.70**	**93.90**	**97.39**	**94.65**

All evaluation metrics are reported as percentages (%). The best results are highlighted in bold.

Looking at the overall results, the baseline model achieved an mIoU of 87.77% without any enhancement modules. Building on this, adding LFM-HFE alone resulted in only a slight improvement in model performance, with mIoU increasing to 87.81%. This indicates that while relying solely on high-frequency information enhancement can improve classification accuracy to some extent, its overall discriminative capability remains limited due to the lack of an effective structural modeling mechanism. When LFM-HFE was further integrated into DG-SSM to form a complete architecture, the model’s performance improved to 87.90%. This indicates that the dual-gating mechanism, which synergistically modulates frequency-domain information and spatial semantics, can more effectively integrate shallow-level details with deep-level semantics, thereby achieving more stable representation capabilities in complex texture regions. When CGFS was introduced independently, the model’s mIoU reached 88.41%, indicating that global semantic constraints can effectively reduce classification confusion between different growth stages and further improve class consistency.

When DG-SSM and CGFS work in concert, the model performance reaches its peak, with mIoU increasing to 88.70% and mF1 reaching 93.90%. These results demonstrate that WaveletUMamba can effectively improve the classification accuracy of celery cultivation areas at different growth stages in complex agricultural scenarios.

**Figure 11:**
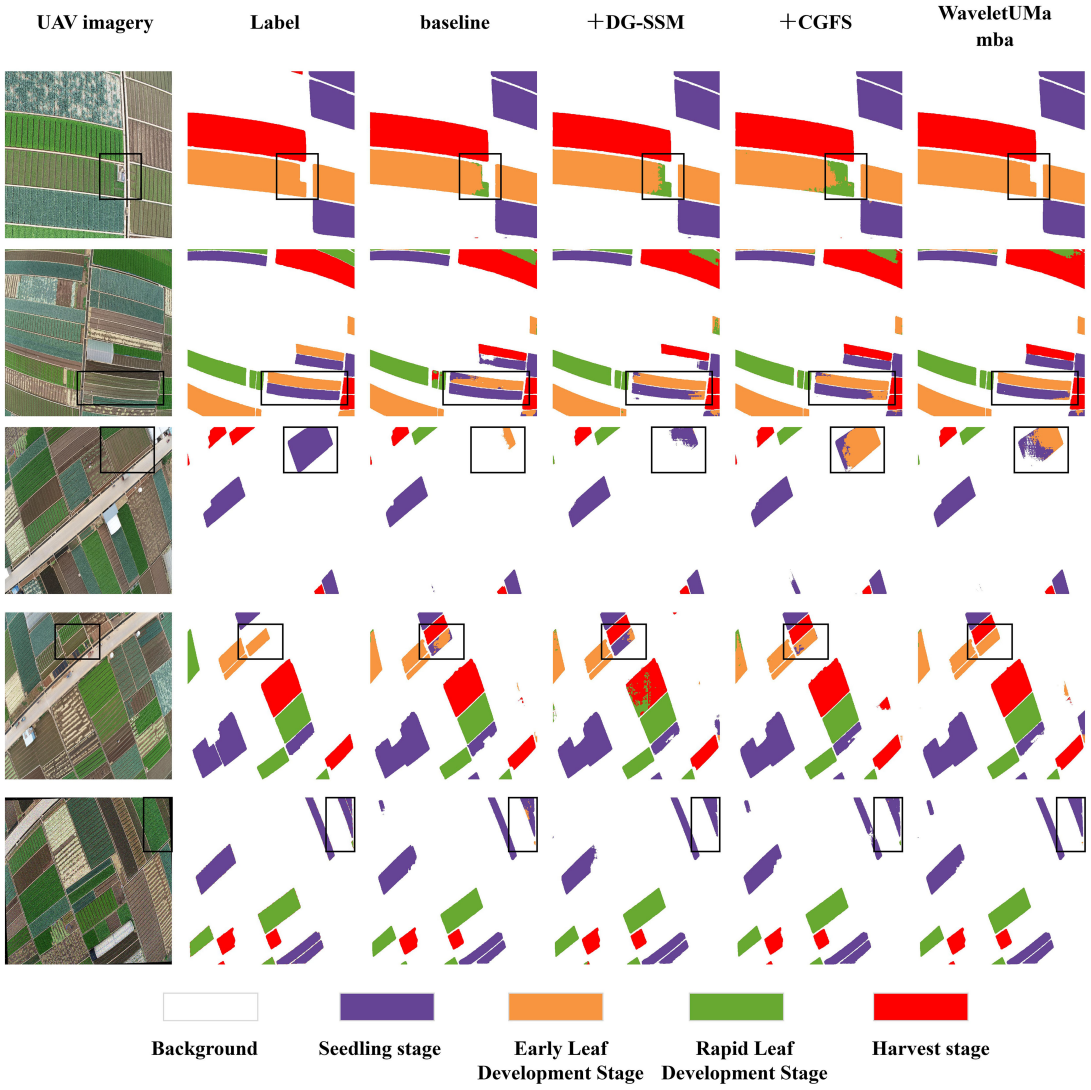
Visual comparison of ablation results. Black boxes emphasize the differences among configurations.

**Figure 11** shows the visualization results of classification under different module combinations. The baseline model is capable of reasonably well restoring the overall spatial structure of farmland parcels, but it still exhibits significant shortcomings in complex scenarios. In transition zones between different growth stages, the model struggles to accurately distinguish class boundaries, leading to confusion between adjacent classes. For complex parcels—such as those that are narrow, elongated, or fragmented—boundary discontinuities and object omissions are common, and misclassification occurs in some local areas, making it difficult to adequately model complex boundaries and fine-grained structural information.

After introducing the DG-SSM module, the model’s responsiveness to high-frequency structures was enhanced. In multiple annotated regions, class boundaries became clearer. However, due to the model’s increased sensitivity to local texture changes, some areas exhibited fragmented responses. While the high-frequency enhancement mechanism improved detail, it may have had some impact on regional consistency. Upon further introduction of the CGFS module, the model demonstrated significant improvement in regional consistency, and the misclassification rate decreased. Compared to using DG-SSM alone, predictions within large areas are more coherent, and local fragmentation is reduced. This indicates that global semantic guidance helps suppress local noise and improve the stability of class discrimination. When both DG-SSM and CGFS are introduced simultaneously, the visualization results show that the model not only maintains clear parcel boundaries but also effectively reduces noise within regions, improving classification accuracy and bringing the prediction results closer to the ground truth in complex scenarios.

Ablation experiments show that DG-SSM focuses on enhancing the representation of high-frequency details, which helps improve boundary delineation, while CGFS improves regional consistency and alleviates category confusion by introducing global semantic constraints. The synergy between the two enables the model to achieve a better balance between fine-grained structural representation and global semantic consistency, thereby effectively improving overall performance.

## 6 Discussion

In complex open-field environments, celery at different growth stages in remote sensing imagery exhibits strong similarity in both spectral signatures and spatial morphology. Meanwhile, factors such as shadow occlusion, exposed soil, and mixed cropping further complicate the scene, often leading to blurred boundaries and inter-class confusion in planting information extraction. To address these issues, this paper proposes a state-space model based on wavelet enhancement, named WaveletUMamba. Experimental results show that this method outperforms existing comparison methods in overall segmentation performance and demonstrates greater stability in identifying complex boundaries and easily confused growth stages.

CNN-based methods can extract local features using multiscale convolutions, but due to their limited receptive fields, they struggle to effectively model large-scale contextual information. Transformer-based methods enhance the ability to model global dependencies through self-attention mechanisms, leading to improvements in overall accuracy, but they incur significant computational overhead. In contrast, the state-space model-based Mamba method strikes a good balance between computational efficiency and global modeling. However, its selective scanning mechanism tends to favor the propagation of low-frequency information, which can easily result in the smoothing of high-frequency details, thereby affecting the ability to represent fine-grained structures. This paper introduces wavelet frequency-domain decomposition to decompose features into different frequency subbands, thereby separating high-frequency edge and texture information from the mixed representation. By coordinating this with the state-space model, it effectively compensates for Mamba’s shortcomings in capturing high-frequency information and, to a certain extent, enhances the model’s ability to represent edge textures and fine-grained structures. The designed context-guided feature supervision module extracts local texture information through local detail modeling and utilizes global context weighting for guidance. Acting as an auxiliary supervision branch, it provides guided supervision for deep features during the decoding stage, further enhancing the model’s ability to recognize complex boundaries and small-scale objects, while alleviating semantic confusion caused by spectral and textural similarities between different growth stages.

Although the proposed method outperforms all competing models in overall performance, with particularly notable improvements for the seedling stage and early leaf development stage, the performance gains for the rapid leaf development stage and harvest stage are relatively limited. During the seedling stage, plants are small and the proportion of exposed soil is high, resulting in abundant boundary information. The early leaf development stage represents a transitional period between adjacent phenological stages and exhibits high spectral and textural similarity with neighboring classes. Both scenarios rely heavily on local high-frequency information for accurate discrimination and therefore benefit more substantially from the proposed frequency-domain enhancement mechanism and the local detail modeling capability of the CGFS module.

In contrast, during the rapid leaf development stage and harvest stage, the plant canopy is largely closed, the target regions exhibit stronger spatial continuity, and the class characteristics become more stable, enabling most models to achieve relatively high segmentation accuracy. Although WaveletUMamba achieves the highest IoU for the harvest stage, its performance on the rapid leaf development stage is slightly lower than that of UNetMamba. This observation suggests that, while enhancing local high-frequency information effectively improves the recognition of challenging classes, it may introduce unnecessary feature perturbations for relatively simple classes with highly stable textures, resulting in slight performance fluctuations. Future work will investigate adaptive frequency-domain modeling strategies that dynamically adjust the contributions of different frequency components according to image content, thereby further enhancing feature representation capability in complex scenarios.

Furthermore, the current experiment is based solely on a multi-growth-stage celery dataset from a single region. The model’s generalization ability across regions, crops, and different imaging conditions has not yet been systematically validated. Future work will be expanded to include high-precision agricultural remote sensing datasets spanning multiple regions, crops, and imaging conditions (Zhang et al., 2026) to conduct a more comprehensive evaluation of the model’s transferability and robustness, thereby enhancing its stability and adaptability in complex agricultural scenarios.

## 7 Conclusion

To address issues such as blurred boundaries and class confusion encountered during the extraction of celery cultivation areas at different growth stages in complex open-field agricultural environments, this paper proposes WaveletUMamba, a refined extraction model that integrates frequency-domain enhancement with state-space modeling. Through the collaborative design of frequency-domain enhancement and state-space modeling, this model strengthens the joint representation of local details and global semantic information. It also introduces a context-guided feature supervision mechanism to further enhance the semantic consistency and class discrimination capability of features during the decoding stage, achieving excellent extraction performance on the drone remote sensing celery dataset collected from open-field farmland in Tonghai County. The main conclusions are as follows:

1. WaveletUMamba achieved superior overall performance among all compared methods—including CNNs, Transformers, and Mamba—and struck a good balance between extraction accuracy and computational efficiency. The model’s mF1, mIoU, OA, and Kappa reached 93.90%, 88.70%, 97.39%, and 94.65%, respectively—representing improvements of 0.30%, 1.07%, 0.53%, and 0.63% over the second-best model, UNetMamba—validating the effectiveness of the proposed method for extraction tasks in celery cultivation areas spanning multiple growth stages.
2. In complex open-field agricultural scenarios, WaveletUMamba demonstrates strong adaptability to complex backgrounds, small-scale targets, and adjacent growth stages. It achieves more significant performance improvements in categories that are difficult to identify, such as the seedling stage and the early leaf development stage, effectively mitigating interference from complex backgrounds and category confusion between adjacent growth stages. At the same time, the model demonstrates better regional consistency and boundary preservation capabilities during the extraction of complex plot boundaries and fine-grained structures. The research results indicate that the proposed method strikes a balance between segmentation accuracy and computational efficiency, providing a reference for the precise extraction of leafy vegetable croplands across multiple growth stages using UAV remote sensing.

## Data Availability

The raw data supporting the conclusions of this article will be made available by the authors, without undue reservation.
